# Affibody-Derived Drug Conjugates Targeting The Epidermal Growth Factor Receptor Are Potent And Specific Cytotoxic Agents

**DOI:** 10.1021/acsptsci.5c00079

**Published:** 2025-10-31

**Authors:** Sara S. Rinne, Wen Yin, Ruonan Li, Haozhong Ding, Anna Mestre Borras, Chenar Mahmod, Stefan Ståhl, Anna Orlova, John Löfblom, Anzhelika Vorobyeva, Torbjörn Gräslund

**Affiliations:** † Department of Medicinal Chemistry, 8097 Uppsala University, 751 23 Uppsala, Sweden; ‡ Department of Protein Science, 7655 KTH Royal Institute of Technology, Roslagstullsbacken 21, 11421 Stockholm, Sweden; § Department of Immunology, Genetics and Pathology, Uppsala University, Dag Hammarskjölds väg 20, 751 85 Uppsala, Sweden

**Keywords:** antibody, ADC, affibody, ABD, DM1, epidermal growth factor receptor, EGFR, cancer

## Abstract

Overactive epidermal growth factor receptor (EGFR) signaling is often involved in driving different types of carcinomas. It is a well-studied target for targeted therapies, with both monoclonal antibodies and kinase inhibitors available for clinical use. Even though these drugs show a clinical benefit, most patients develop resistance over time. The development of new therapeutic modalities is therefore highly motivated. Herein, we describe a new type of drug candidate targeting EGFR, a so-called affibody-based drug conjugate. It consists of an EGFR-targeting affibody molecule, Z_EGFR_, expressed as a fusion to an albumin-binding domain for half-life extension, and coupled with the potent cytotoxic drug DM1 via a maleimidocaproyl linker. The resulting drug conjugate Z_EGFR_-ABD-mcDM1, showed strong binding to recombinant EGFR and EGFR-expressing cells. It was found to be highly potent in killing EGFR-expressing A431 cells with an IC_50_ of 3.4 nM. *In vivo*, it showed moderate uptake in A431-derived xenografts with high EGFR expression. Collectively, the results from this study, demonstrate a potent and EGFR-specific drug candidate that holds promise for further development.

## Graphical abstract

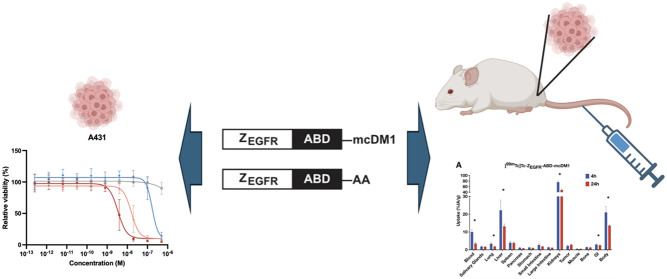

Targeted drugs have been introduced in clinical practice and have transformed cancer treatment profoundly. One class of targeted drugs is the monoclonal antibodies (mAbs), which may directly target receptors overexpressed on cancer cells to inhibit their function and invoke antitumor immune reactions.^1^ Another class is the antibody-drug conjugates (ADCs), which consist of mAbs conjugated with potent cytotoxic drugs.^2^ While they may also inhibit the function of the targeted receptor, and invoke an immune response, the main therapeutic effect comes from the cytotoxic action of the attached drug. A third class of targeting drugs used in cancer therapy is the kinase inhibitors.^3^ Many of them have shown potent efficacy. In comparison to mAbs, they are usually less specific in their targeting.

The epidermal growth factor receptor (EGFR, ErbB1) belongs to the ErbB family of receptor tyrosine kinases.^4^ It is involved in the maintenance of epithelial tissue homeostasis. There are several known ligands that can activate EGFR, including epidermal growth factor (EGF), transforming growth factor (TGF)-α, epigen (EPG), β-cellulin (BTC), heparin binding EGF (HB-EGF), and epiregulin (EPR).^5^ Upon activation, EGFR forms homo- or heterodimers with other members of the ErbB family, leading to cross-phosphorylation of the receptors’ intracellular domains. The dimers can then, in turn, directly or indirectly activate downstream signaling through, for example, the mitogen-activated protein kinase (MAPK),^6^ leading to cell growth and suppression of apoptosis.

Altered EGFR function is involved in the development and maintenance of many different carcinomas, for example, non-small cell lung cancer (NSCLC), glioblastoma multiforme (GBM), breast cancer (BC), colorectal cancer (CRC) and head and neck squamous cell carcinoma (HNSCC).^7–10^ It becomes overactive as a result of overexpression, mutations, or deletions.^10^

The understanding of the central role of EGFR in many carcinomas has led to the development of different types of EGFR-targeted drugs. The tyrosine kinase inhibitors (TKIs), gefitinib and erlotinib, are two drugs approved for clinical use in patients with NSCLC. These first-generation TKIs have shown impressive efficacy for patients with advanced NSCLC with mutant EGFR.^11,12^ However, for essentially all of the patients, resistance develops over time, most commonly as a consequence of a T790M mutation in EGFR.^13^ Second and third-generation TKIs have also been developed, for subsequent use when patients have developed resistance. They have also shown clinical benefits, but most patients eventually develop resistance to those compounds as well.^14^

Cetuximab is an EGFR targeting mAb that competes with EGF for binding to the receptor. It is used clinically for the treatment of CRC and HNSCC. Panitumumab is another EGFR targeting mAb that is used for the treatment of CRC. Both mAbs have shown a clinical benefit, however, most patients develop resistance to these drugs within months.^15^

ADCs targeting EGFR are under clinical development, but no drug is yet approved for clinical use. Depatuxizumab mafodotin is an ADC consisting of an anti-EGFR mAb with a monomethyl auristatin F (MMAF) payload.^16^ It has undergone clinical testing on patients with GBM. Early clinical results were promising, but the phase III INTELLANCE-1 study failed to meet the clinical endpoint of increased patient survival.^17^ Another ADC that underwent a phase I clinical trial is losatuxizumab vedotin. While it showed promising results, infusion-related adverse events of an unknown source led to premature termination of the trial.^18^

A complicating factor during the development of EGFR-targeted drugs is that some normal tissues express EGFR, e. g. the liver,^19^ which could lead to on-target/off-tumor uptake. Also, due to its expression in the skin, dermatological side effects are common in patients treated with EGFR-targeted drugs.^20^

Even though the currently available anti-EGFR drugs may give a clinical benefit to patients, they are rarely curative. It is therefore motivated to investigate new EGFR-targeting modalities that may be superior or that can complement the currently available drugs.

Herein, we have designed and characterized a new type of EGFR-targeted drug conjugate derived from a so-called affibody molecule. Affibody molecules, comprising 58 amino acids, are folded peptides derived from the B-domain of protein A from *Staphylococcus aureus*.^21^ By randomization of 13 amino acids located in helix one and two, combinatorial libraries have been created. From these libraries, affibody molecules binding to many different targets have been selected.^21^ Affibody molecules with strong and specific affinity to EGFR have been described earlier.^22–24^ In particular, the affibody molecule Z_EGFR:2377_ interacts specifically with human and mouse EGFR, and with similar affinity (0.8–0.9 nM).^24,25^ An advantage of using affibody molecules compared to mAbs for the delivery of cytotoxic drugs is their smaller size which should lead to better tumor penetration and more efficient drug delivery to the tumor’s interior.^26^

Different types of cytotoxic drugs have been used for the creation of drug conjugates. The maytansinoids are a type of extremely potent cytotoxic drugs that have been found suitable in several ADCs under clinical development as well as in the clinically approved trastuzumab emtansine (T-DM1), which targets human epidermal growth factor receptor 2 (HER2).^27^ In T-DM1, the drug is attached to the mAb via a noncleavable linker. After T-DM1 binds to HER2 on the cell surface, it is endocytosed and transported to the lysosomes where the protein part of T-DM1 is degraded. The released DM1, along with the linker and the lysine to which it was conjugated in the mAb, can then enter the cytosol to exert its toxic function. DM1 kills the cells by binding to tubulin, to disrupt microtubule formation.

Since affibody molecules are small, they are cleared rapidly from circulation by filtration in the kidneys. A way to increase the half-life in circulation and increase the bioavailability is fusion to an albumin-binding domain (ABD). Upon injection, the ABD associates with albumin, which increases the size of the complex by 67 kDa, to above the cutoff for kidney filtration. Moreover, binding to albumin prolongs the half-life further due to albumin’s interaction with FcRn, which prevents lysosomal degradation by e.g., cells in contact with blood. A well-explored ABD is the G148-GA3 domain from streptococcal protein G, and its engineered progeny ADB_035_, with a femtomolar affinity for human serum albumin.^28^

Affibody-based drug conjugates targeting the HER2 receptor have previously been studied. They consisted of an affibody molecule with strong and specific affinity for the HER2 receptor, Z_HER2:2891_, loaded with DM1 or MMAE, and have been found to be specific in their targeting of HER2 overexpressing cells, and potent cytotoxic compounds in in vitro experiments.^29–33^ Z_HER2:2891_ has also been fused to an albumin binding domain (ABD_035_) for *in vivo* half-life extension, and DM1 connected by a noncleavable maleimidocaproyl (mc) linker, attached to a cysteine amino acid placed in the C-terminus of the fusion protein. This construct was shown to allow for efficient treatment of xenografted HER2-overexpressing ovarian tumors in mice.^32,33^ A study concerning the order and multiplicity of affibody domains fused to the ABD and loaded with DM1 showed that the preferred architecture was an N-terminal affibody followed by a C-terminal ABD.^34^ Furthermore, in the first in vivo experiments on affibody-based drug conjugates, an unexpectedly high uptake in the liver was observed.^32^ Liver uptake should preferably be minimized since liver toxicity is a common side effect of many protein drugs. It is of particular importance when developing drugs targeting EGFR since the liver has endogenous EGFR expression,^35^ which further increases the uptake. Moreover, the second most common grade 3 or higher adverse event for patients receiving T-DM1 is elevated liver enzymes in the blood,^36^ pointing toward liver damage. In an earlier study on affibody-drug conjugates, it was found that the liver uptake could be reduced by the addition of a linker sequence, consisting of the amino acids Glu-Glu-Glu, next to the C-terminal cysteine to which DM1 was conjugated.^37^ It is likely that the negative charge and hydrophilic nature of the glutamic acids shield the hydrophobic DM1 from nonspecific uptake by scavenger receptors in the liver.

In this study, we have capitalized on the knowledge generated in the previous studies on drug conjugates targeting HER2 when creating a drug conjugate targeting EGFR. The EGFR-targeted conjugate consisted of an N-terminal affibody, Z_EGFR:2377_, followed by ABD_035_, a Glu-Glu-Glu linker, and a C-terminal cysteine to which the cytotoxic DM1 drug was conjugated via a maleimidocaproyl (mc) linker (Figure 1). An extension with the amino acid sequence His-Glu-His-Glu-His-Glu (a (HE)_3_-tag) was placed in the N-terminus, which was used for radiolabeling with 99m-Technetium to allow tracking *in vivo*.^38,39^ The HE_3_-tag was used since it has been shown not to affect liver uptake, in contrast to the more commonly used hexahistidine tag, which increases uptake in the liver.^40^ The drug conjugate was characterized biochemically, and its biodistribution in mice was determined to investigate the suitability of using an affibody molecule for targeted delivery of DM1 to EGFR-expressing tumors.

**Figure 1:**
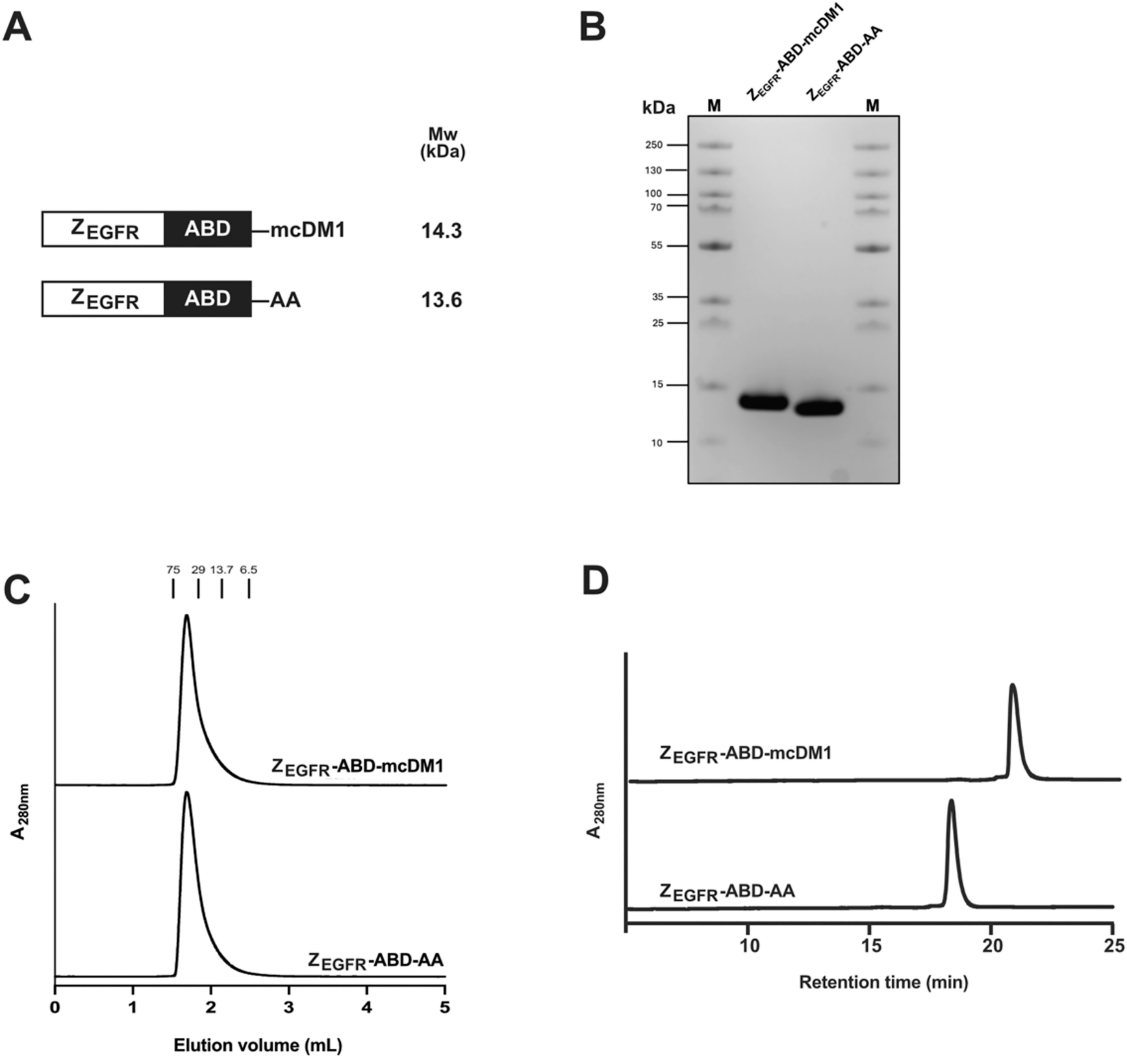
Schematic representation and primary characterization of the drug conjugate Z_EGFR_-ABD-mcDM1 and the nontoxic control Z_EGFR_-ABD-AA. (A) The composition of the two constructs with theoretical molecular weights (Mw). (B) The purified constructs (10 μg/sample) were analyzed by SDS-PAGE under reducing conditions. The numbers to the left of the panel indicate the molecular weights of marker proteins in the M lanes. (C) Chromatograms obtained after analysis by size-exclusion chromatography. The numbers above the panel indicate the molecular weights of protein standards. (D) Chromatograms obtained from the analysis by RP-HPLC. The constructs were eluted with a 30 min linear gradient from 30–60% acetonitrile in water supplemented with 0.1% trifluoroacetic acid.

## 2 Materials and Methods

All chemicals and kits were from Thermo Fisher Scientific (Waltham, MA) or Merck (Kenilworth, NJ) unless otherwise indicated.

### 2.1 Gene Design, Expression, and Purification

The affibody molecule Z_EGFR:2377_,^24^ targeting EGFR, was used in this study, hereafter abbreviated Z_EGFR_. The albumin binding domain, ABD_035_,^28^ hereafter abbreviated ABD, was used in this study. The genes encoding Z_EGFR_ and ABD were subcloned for cytoplasmic expression into the expression plasmid pET26b by in-fusion cloning following the protocol recommended by the manufacturer (Takara, Kusatsu, Japan), yielding a gene encoding Z_EGFR_-ABD. During subcloning, a sequence encoding the amino acids Met-His-Glu-His-Glu-His-Glu was added to the 5′-end of the gene, and a sequence encoding the amino acids Glu-Glu-Glu-Cys was added to the 3′-end. The integrity of the gene construct was verified by DNA sequencing (Eurofins genomics, Ebersberg, Germany).

Z_EGFR_-ABD was expressed in *Escherichia coli* (*E. coli*) BL21 Star (DE3) (New England Biolabs, Ipswitch, MA). An overnight culture was diluted into cultivation medium (30 g/L Tryptic soy broth supplemented with 5 g/L yeast extract and 50 μg/mL kanamycin) at a 1:100 ratio, followed by cultivation at 37 °C until OD_600_ had reached 0.5. Protein expression was induced by adding isopropyl β-d-1-thiogalactopyranoside (IPTG) to a final concentration of 1 mM, followed by cultivation for 16 h at 25 °C. The cells were then harvested by centrifugation and subsequently resuspended in loading buffer (25 mM Tris, 1 mM EDTA, 200 mM NaCl, 0,05% Tween 20, pH 8.0), supplemented with 1% complete EDTA-free protease inhibitor cocktail (Roche Diagnostic, Basel, Switzerland). The cells were disrupted by French Pressing, after which the supernatant of the lysed cells was collected after centrifugation. Z_EGFR_-ABD was purified by affinity chromatography using HSA as the immobilized ligand on the column, essentially as described.^41^ The eluted proteins were pooled and lyophilized.

### 2.2 Conjugation with mcDM1

The lyophilized Z_EGFR_-ABD was reconstituted in PBS (pH 6.8) to a final concentration of 0.1 mM. To reduce potentially oxidized cysteines, the protein was incubated with freshly dissolved tris­(2-carboxyethyl) phosphine (TCEP) at a final concentration of 5 mM. Thereafter, maleimidocaproyl-DM1 (mcDM1) (Levena Biopharma, San Diego, CA) was added to a final concentration of 0.25 mM, and conjugation was carried out for 1 h at room temperature. The reaction mixture was passed through a PD-10 desalting column to remove unreacted mcDM1 and TCEP. The eluted material from the PD-10 column was further purified by reversed-phase high-performance liquid chromatography (RP-HPLC) using a Zorbax C18 SB column (Agilent, Santa Clara, CA). Elution was with a linear gradient (1%/min) between 25% and 60% acetonitrile in water with 0.1% trifluoroacetic acid (TFA). The flow rate was 3 mL/min. The fractions containing Z_EGFR_-ABD-mcDM1 were pooled and lyophilized.

In parallel, a nontoxic control, Z_EGFR_-ABD-AA, was prepared by alkylation of the cysteine in Z_EGFR_-ABD. The lyophilized fusion protein was reconstituted in 200 mM ammonium bicarbonate (pH 8.0) supplemented with 2% sodium dodecyl sulfate to a final concentration of 1 mM. Thereafter, TCEP was added to a final concentration of 5 mM to reduce potentially oxidized cysteines. Freshly prepared 2-iodoacetamide was used to alkylate the cysteine in Z_EGFR_-ABD. It was added to Z_EGFR_-ABD to a final concentration of 20 mM, followed by incubation for 30 min in the dark at room temperature. Z_EGFR_-ABD-AA was purified by RP-HPLC as described above for Z_EGFR_-ABD-mcDM1.

### 2.3 Analysis by RP-HPLC, Size-Exclusion Chromatography, and Circular Dichroism Spectroscopy

Z_EGFR_-ABD-mcDM1 and Z_EGFR_-ABD-AA were analyzed by RP-HPLC. Approximately 4 μg of each construct was dissolved in water supplemented with 0.1% TFA and loaded onto a Zorbax 300SB-C18 column. Elution was carried out with a linear gradient from 30% to 60% acetonitrile in water with 0.1% TFA over 30 min. The flow rate was 1 mL/min.

Z_EGFR_-ABD-mcDM1 and Z_EGFR_-ABD-AA were also analyzed by size-exclusion chromatography. The constructs (4 μg each) was loaded on a prepacked Superdex-75 5/150 column (Cytiva, Uppsala, Sweden) equilibrated with PBS. The samples were subsequently eluted with PBS, at a flow rate of 0.45 mL/min.

Analysis by circular dichroism (CD) spectroscopy was carried out on a Chirascan spectropolarimeter (Applied Photophysics, Leatherhead, UK). The constructs were dissolved in water to 0.4 mg/mL and analyzed in a 1 mm cuvette. For analysis of secondary structure content, the wavelength was varied from 260 to 195 nM. Thermal denaturation was carried out by increasing the temperature from 20 to 95 °C while recording the ellipticity at 221 nm. The highly α-helical Z_EGFR_-ABD-mcDM1 and Z_EGFR_-ABD-AA constructs have a large negative ellipticity at this wavelength when folded.

### 2.4 Analysis by Surface Plasmon Resonance

The interaction of Z_EGFR_-ABD-mcDM1 and Z_EGFR_-ABD-AA with the extracellular domains of human EGFR (hEGFR), and mouse EGFR (mEGFR) was investigated on a Biacore 8K instrument (Cytiva). HSA was immobilized by amine coupling on a CM5 chip. A blank surface was created by activating and deactivating a flow cell on each channel. Next, Z_EGFR_-ABD-mcDM1 and Z_EGFR_-ABD-AA (100 nM) were captured over the different channels, followed by injection of hEGFR or mEGFR at different concentrations (3.1–100 nM, except for hEGFR at 25 °C where 3.1–50 nM was used). The binding analyses were carried out at 25 or 37 °C with PBS supplemented with 0.05% Tween-20 (pH 7.4) as running buffer at the flow rate of 30 mL/min. The chip was regenerated by injection of 10 mM glycine-HCl (pH 2.0) for 30 s at a flow rate of 50 mL/min. The kinetic constants of the interactions were derived with the Biacore software assuming a 1:1 Langmuir interaction.

### 2.5 Cell Culture

The cell lines A431 (skin cancer), H292 (lung cancer), DU145 (prostate cancer), T47D (breast cancer), MCF7 (breast cancer) and PC-3 (prostate cancer) were obtained from ATCC (Manassas, VA). The cells were grown in RPMI 1640 media (Biowest, Riverside, MO) supplemented with 300 mg/L of l-Glutamine, 10% fetal bovine serum, and 1% penicillin/streptomycin in a humidified incubator with 5% CO_2_ at 37 °C. A transcriptome analysis of mRNA encoding EGFR in a collection of cell lines is displayed on www.proteinatlas.org.^42^ According to the analysis, the A431 cell line has a normalized transcript expression value (nTPM) of 2978, the H292 cell line has a nTPM of 87, the DU145 has a nTPM of 55, PC-3 has a nTPM of 46, the T47D cell line has a nTPM of 3, and the MCF-7 cell line has a nTPM of 1.4.

### 2.6 Analysis of Cytotoxicity

To evaluate the cytotoxic potential of Z_EGFR_-ABD-mcDM1, free mcDM1, and the nontoxic control Z_EGFR_-ABD-AA, one cell line with high expression of EGFR (A431), two cell lines with medium/high expression of EGFR (H292 and DU145), one cell line with medium expression of EGFR (T47D), and one cell line with low EGFR-expression (MCF-7) were used. The cells were seeded at 5000 cells/well in a 96-well plate and were allowed to attach overnight. On the following morning, Z_EGFR_-ABD-mcDM1, mcDM1, or Z_EGFR_-ABD-AA were added to the wells at different concentrations. For the blocking experiment, the cells were first preincubated with Z_EGFR_-ABD-AA (1000 nM) for 30 min at room temperature, followed by removal of the blocking solution and addition of Z_EGFR_-ABD-mcDM1 at different concentrations. The cells were incubated for 72 h, after which the viability was measured by using the Cell Counting Kit-8 (Sigma-Aldrich, St. Louis, MO) following the recommended protocol from the manufacturer.

### 2.7 Radiolabeling with [^99m^Tc]Tc and Analysis of Labeling Stability

Z_EGFR_-ABD-mcDM1 and Z_EGFR_-ABD-AA were labeled with [^99m^Tc]­Tc­(CO)_3_ on the N-terminal (HE)_3_-tag.^38^ [^99m^Tc]Tc in the form of [^99m^Tc]­TcO_4_ was obtained by elution from an Ultra-TechneKow generator (Mallinckrodt, Staines-upon-Thames, United Kingdom) with sterile 0.9% sodium chloride. [^99m^Tc]­TcO_4_ was converted to [^99m^Tc]­Tc­(CO)_3_ using a CRS kit (Paul Scherrer Institute, Villingen, Switzerland) according to a previously published protocol.^38,43^ For labeling, 50 μg of Z_EGFR_-ABD-mcDM1 or Z_EGFR_-ABD-AA, dissolved in 41 μL of PBS were mixed with 40 μL of [^99m^Tc]­Tc­(CO)_3_ (280–380 MBq) from the CRS kit and incubated for 60 min at 50 °C. The radiochemical yield was determined by radio-ITLC. For this, a sample of the radiolabeling mixture was applied to silica gel-impregnated glass microfiber chromatography paper (Agilent Technologies), which was eluted with PBS. The activity distribution over the paper was analyzed using the Cyclone storage phosphor system and OptiQuant image analysis software (PerkinElmer, Waltham, MA). The presence of reduced hydrolyzed technetium colloids (RHT) was determined by ITLC using a solution of pyridine:acetic acid:water (5:3:1.5) for elution. The labeled constructs were purified using NAP5 size exclusion columns (Cytiva) according to the manufacturer’s instructions. The purity was determined by radio-ITLC as described above.

To assess the *in vitro* stability of the radiolabeled constructs, 1 μg of the purified radiolabeled constructs was incubated in 50 μL of PBS at room temperature, or 50 μL of PBS containing a 1000-fold molar excess of histidine at room temperature or at 37 °C. Samples were collected after 1, 4, and 24 h of incubation, and the amount of protein-associated activity was determined by ITLC.

### 2.8 Cell Binding Specificity, Internalization, and Cellular Retention

To test the EGFR-binding specificity, EGFR expressing A431, H292, DU145, and PC-3 cells were incubated with either 10 nM (A431, H292) or 5 nM (DU145, PC-3) of the radiolabeled constructs for 1 h at 37 °C. Prior to the addition of the radiolabeled constructs, half of the dishes were incubated with 1000 nM cetuximab, which competes with Z_EGFR_ for binding to EGFR, for 15 min to block available EGFR. After incubation, the cells were collected, and the activity content was measured in an automated γ counter.

Internalization of [^99m^Tc]­Tc-Z_EGFR_-ABD-mcDM1 was studied in A431, H292, PC-3, and DU145 cells according to a previously published protocol.^44^ In brief, the cells were incubated with 1 nM of [^99m^Tc]­Tc-Z_EGFR_-ABD-mcDM1 at 37 °C. After 1, 2, 4, 6, and 24 h, a set of dishes was collected, and the membrane-bound and internalized activities were determined using the ‘acid wash’ method.^44^

### 2.9 Cell Binding Analysis

The binding kinetics of [^99m^Tc]­Tc-Z_EGFR_-ABD-mcDM1 and [^99m^Tc]­Tc-Z_EGFR_-ABD-AA to living A431 cells were studied in real-time using a LigandTracer yellow instrument (Ridgeview Instruments, Uppsala, Sweden). The cells were seeded in a predefined area of a 10 cm Petri dish (3 × 10^6^ cells/dish) and were allowed to attach overnight. On the next day, the dish was placed in the inclined rotating holder of the LigandTracer instrument, and medium containing a radiolabeled construct was added at increasing concentrations. The association was measured at 1 and 3 nM, where 3 nM was added once the 1 nM concentration had reached equilibrium. To analyze the dissociation, the radioactive solution was replaced with a medium lacking any construct. The association rate (*k*_a_), dissociation rate (*k*_d_), and equilibrium dissociation constant (*K*_D_) were determined using TraceDrawer (Ridgeview Instruments) assuming a 1:1 interaction model.

### 2.10 Biodistribution in Mice

The animal experiments were approved by the ethics committee for animal research in Uppsala (Sweden, approval number 473/21). Female BALB/c nu/nu mice were inoculated with EGFR-expressing A431 cells 10 days prior to the start of the experiment. Control mice were inoculated with EGFR-negative RAMOS cells 20 days prior to the start of the experiment. The biodistribution was determined at 4 and 24 h after intravenous injection of 1.9 μg (0.06–0.64 MBq) of [^99m^Tc]­Tc-Z_EGFR_-ABD-mcDM1 per mouse (*n* = 4 for each time point). At selected time points, the mice were injected intraperitoneally with ketamine (250 mg/kg) and xylazine (25 mg/kg) and sacrificed by heart puncture. The blood, salivary glands, lungs, liver, spleen, pancreas, stomach, small intestine, large intestine, kidneys, tumors, muscle, and bone were collected and weighed. The radioactivity content in the samples was measured using an automated γ counter (1480 Wizard, Wallac, Finland). Unpaired, two-tailed Student’s *t* tests were used to determine statistical differences (*p* < 0.05) using Prism (GraphPad Software, La Jolla, CA).

## 3 Results

### 3.1 Production and Primary Characterization

The recombinant protein, Z_EGFR_-ABD, was expressed in a soluble form in *E. coli*, typically with a yield exceeding 10 mg/L. The ABD was used for purification by affinity chromatography on a resin with immobilized human serum albumin (HSA). Subsequently, mcDM1 was conjugated to a cysteine placed in the C-terminus, yielding Z_EGFR_-ABD-mcDM1 (Figure 1A). A nontoxic control construct was also generated by alkylation of the C-terminal cysteine, giving Z_EGFR_-ABD-AA. After further purification, samples were analyzed by SDS-PAGE (Figure 1B), which showed highly pure products with essentially the correct molecular weight. The constructs were analyzed by size-exclusion chromatography under native conditions (Figure 1C). They were eluted slightly early as single, relatively symmetrical peaks, suggesting monodisperse constructs and the absence of degradation products. The constructs were also analyzed by reversed-phase high-performance liquid chromatography (RP-HPLC) and were eluted as single peaks (Figure 1D). Calculation of the area under the curve showed that the constructs were >99% pure. Elution from the column was carried out by an increasing gradient of acetonitrile in water, resulting in a mobile phase with increasing hydrophobic character. Z_EGFR_-ABD-mcDM1 was eluted later than Z_EGFR_-ABD-AA, showing that it was more hydrophobic, a consequence of the attached mcDM1. The constructs were analyzed by mass spectrometry, and the recorded molecular weights agreed within 1 Da with the theoretical values, further supporting correct and homogeneous constructs (Figure S1).

### 3.2 Characterization by Circular Dichroism Spectroscopy

To characterize the unfolding and refolding of the constructs, they were subjected to heat-induced denaturation (Figure 2A,C). Since both Z_EGFR_ and the ABD are highly α-helical, the unfolding was followed at 221 nm since helices have a large negative ellipticity at this wavelength. The data showed relatively flat transitions to the unfolded state with melting temperatures of 53 and 54 °C for Z_EGFR_-ABD-mcDM1 and Z_EGFR_-ABD-AA, respectively. Variable wavelength scans of both constructs, both before and after heat-induced denaturation, showed a high level of α-helicity (Figure 2B,D), suggesting that expression of Z_EGFR_ and the ABD in the same fusion protein did not negatively affect their ability to fold into α-helical structures. Moreover, the highly α-helical spectra of Z_EGFR_-ABD-mcDM1 suggest that the conjugation of DM1 did not negatively affect the ability of Z_EGFR_ or ABD to fold. For both constructs, the scans before and after heat-induced denaturation essentially overlapped, suggesting complete refolding after denaturation.

**Figure 2:**
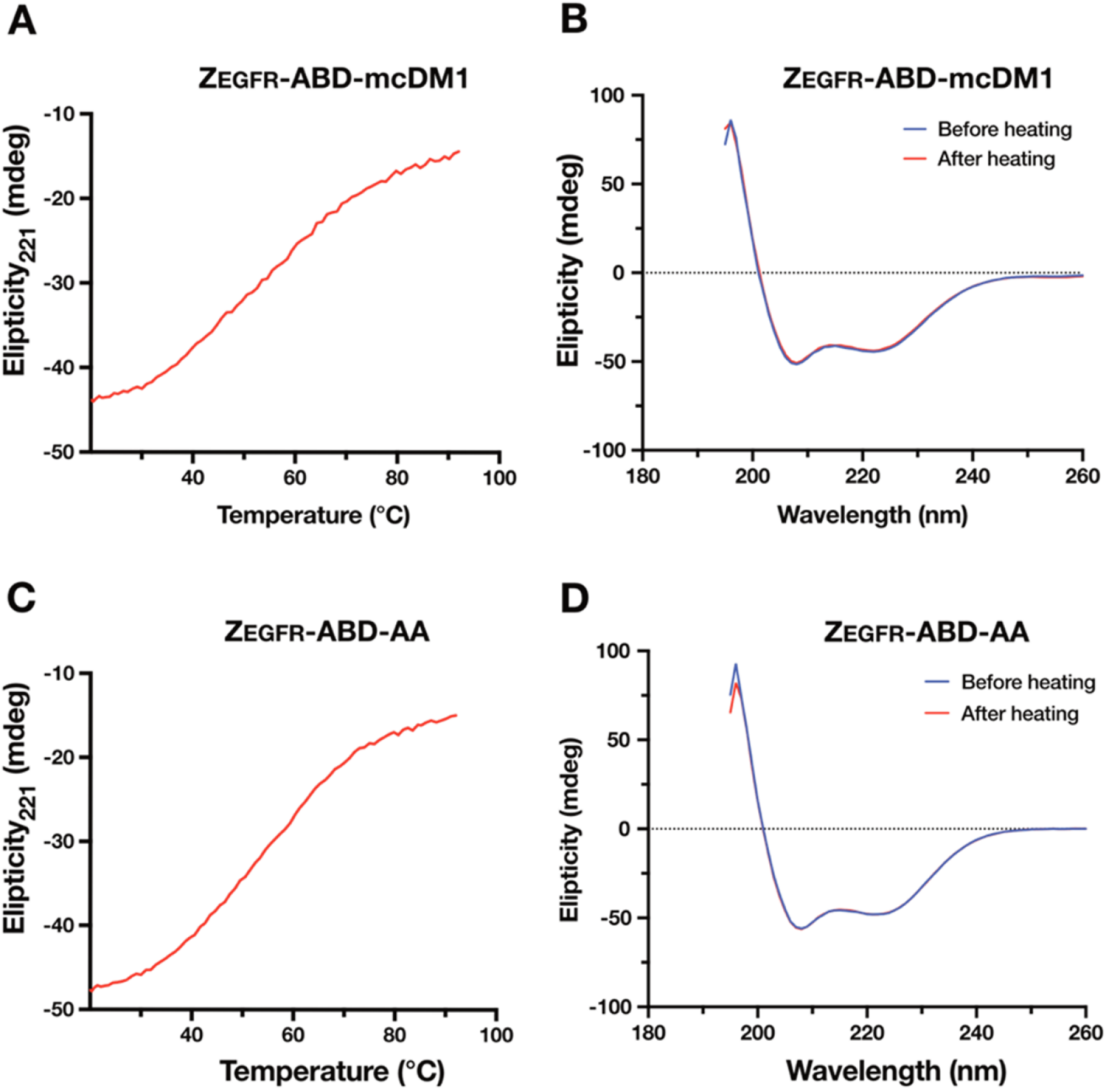
Analysis by circular dichroism spectroscopy. Thermal denaturation of Z_EGFR_-ABD-mcDM1 (A) and Z_EGFR_-ABD-AA (C) was carried out by increasing the temperature from 20 to 90 °C while recording the ellipticity at 221 nm. Panels (B) and (D) show the recorded ellipticity of Z_EGFR_-ABD-mcDM1, and Z_EGFR_-ABD-AA, respectively, while varying the wavelength from 260 to 195 nm.

### 3.3 Determination of the Affinity to EGFR and Serum Albumin

The affinity to the extracellular domain of EGFR was determined by surface plasmon resonance analysis on a Biacore instrument (Table 1, Figure S2 and S3). At 25 °C, the affinities of Z_EGFR_-ABD-mcDM1 and Z_EGFR_-ABD-AA were 2 nM for hEGFR. The affinities for mEGFR were 1 and 2 nM, respectively. These results show that attachment of mcDM1 to the C-terminal cysteine does not affect the affinity to human or mouse EGFR. Moreover, the affinity to the mouse and human orthologs was essentially the same, which suggests that *in vivo* studies on xenografted human tumors in mice could give a relatively correct picture of the on-target off-tumor uptake in mice. At 37 °C, the affinities were marginally weaker. The affinities of Z_EGFR_-ABD-mcDM1 and Z_EGFR_-ABD-AA were 7 and 6 nM for hEGFR, respectively. Both constructs showed affinities to mEGFR of 5 nM at 37 °C. At both temperatures, for both the human and the murine orthologs, material that had been subjected to heat-induced denaturation followed by refolding was also used for affinity determination. In all cases, the determined affinities were essentially the same, further suggesting efficient and complete refolding after heat-induced denaturation.

**Table 1:** Affinities between the Constructs and hEGFR and mEGFR^a^

ligand	temp. (°C)	analyte	*k*_a_ (1/Ms)	*k*_d_ (1/s)	*K*_D_ (nM)
Z_EGFR_-ABD-mcDM1	25	hEGFR	1.4 × 10^5^	2.3 × 10^–4^	1.6
Z_EGFR_-ABD-mcDM1^b^	25	hEGFR	1.3 × 10^5^	2.6 × 10^–4^	2.0
Z_EGFR_-ABD-AA	25	hEGFR	1.4 × 10^5^	2.7 × 10^–4^	1.9
Z_EGFR_-ABD-AA^b^	25	hEGFR	2.5 × 10^5^	1.8 × 10^–4^	0.7
Z_EGFR_-ABD-mcDM1	37	hEGFR	2.6 × 10^5^	1.8 × 10^–3^	6.8
Z_EGFR_-ABD-mcDM1^b^	37	hEGFR	2.9 × 10^5^	2.0 × 10^–3^	6.9
Z_EGFR_-ABD-AA	37	hEGFR	3.4 × 10^5^	2.1 × 10^–3^	6.3
Z_EGFR_-ABD-AA^b^	37	hEGFR	4.0 × 10^5^	2.3 × 10^–3^	5.7
Z_EGFR_-ABD-mcDM1	25	mEGFR	1.5 × 10^5^	2.2 × 10^–4^	1.5
Z_EGFR_-ABD-mcDM1^b^	25	mEGFR	1.6 × 10^5^	2.2 × 10^–4^	1.4
Z_EGFR_-ABD-AA	25	mEGFR	1.4 × 10^5^	2.8 × 10^–4^	2.0
Z_EGFR_-ABD-AA^b^	25	mEGFR	1.6 × 10^5^	2.6 × 10^–4^	1.6
Z_EGFR_-ABD-mcDM1	37	mEGFR	2.2 × 10^5^	1.2 × 10^–3^	5.2
Z_EGFR_-ABD-mcDM1^b^	37	mEGFR	2.4 × 10^5^	1.2 × 10^–3^	4.9
Z_EGFR_-ABD-AA	37	mEGFR	2.2 × 10^5^	1.2 × 10^–3^	5.4
Z_EGFR_-ABD-AA^b^	37	mEGFR	2.6 × 10^5^	1.2 × 10^–3^	4.6

a The kinetic constants were derived from the sensorgrams in Figures S2 and S3, assuming a 1:1 Langmuir interaction.

b Measurement after heat-induced denaturation and renaturation.

The affinities to human and mouse serum albumin (HSA and MSA) were also determined by surface plasmon resonance analysis (Table 2, Figure S4). The affinity of Z_EGFR_-ABD-mcDM1 was essentially the same for both HSA and MSA (3.2 and 3.6 nM, respectively). The affinity of Z_EGFR_-ABD-AA was slightly stronger (0.7 and 0.6 nM, respectively), indicating a slight blocking of the ability to bind serum albumin by conjugation of mcDM1.

**Table 2:** Affinities between the Constructs and HSA and MSA^a^

analyte	temp. (°C)	ligand	*k*_a_ (1/Ms)	*k*_d_ (1/s)	*K*_D_ (nM)
Z_EGFR_-ABD-mcDM1	25	HSA	1.6 × 10^5^	5.2 × 10^–4^	3.2
Z_EGFR_-ABD-AA	25	HSA	6.2 × 10^5^	4.6 × 10^–4^	0.7
Z_EGFR_-ABD-mcDM1	25	MSA	2.4 × 10^6^	8.6 × 10^–3^	3.6
Z_EGFR_-ABD-AA	25	MSA	1.4 × 10^7^	8.4 × 10^–3^	0.6

a The kinetic constants were derived from the sensorgrams in Figure S4, assuming a 1:1 Langmuir interaction.

As a control, Z_EGFR_-ABD-mcDM1 and Z_EGFR_-ABD-AA were also injected over a surface with immobilized HER2 (Figure S5). As expected, no binding was observed to this surface for any of the concentrations injected (up to 200 nM).

### 3.4 *In Vitro* Cytotoxicity

The *in vitro* cytotoxic efficacy of Z_EGFR_-ABD-mcDM1 was investigated on human cell lines with different expression levels of EGFR (Figures 3, S6). The most potent response was found on the A431 cell line (high EGFR expression), where Z_EGFR_-ABD-mcDM1 showed an IC_50_ value of 3.4 nM. For the medium/high EGFR-expressing cell lines H292 and DU145, the IC_50_ values were 3.7 nM and 22 nM, respectively. The IC_50_ value for T47D (medium EGFR expression) was 34 nM. MCF-7 (low EGFR-expression) was unaffected by Z_EGFR_-ABD-mcDM1 up to the highest concentration tested (500 nM). The results show that a higher expression of EGFR leads to a more potent killing effect, suggesting that the killing is EGFR-mediated. Furthermore, the cell lines appear to have a difference in their natural resistance to DM1-killing since there is a difference in the IC_50_ value of A431, DU145 and H292, even though they have a similar expression of EGFR. To investigate this hypothesis, the cell lines were incubated with dilution series of free mcDM1 to determine the sensitivity to the drug. The IC_50_ values were >10 nM for all cell lines with A431 being the most sensitive, followed by T47D, H292, MCF-7, and DU145. The result showed that indeed the sensitivity to Z_EGFR_-ABD-mcDM1 follows the sensitivity to mcDM1 for all cell lines with medium or higher EGFR-expression, suggesting that a certain EGFR-density on the cell surface is required for cell killing, but at that density or higher, the sensitivity to DM1 determines the sensitivity to Z_EGFR_-ABD-mcDM1.

**Figure 3:**
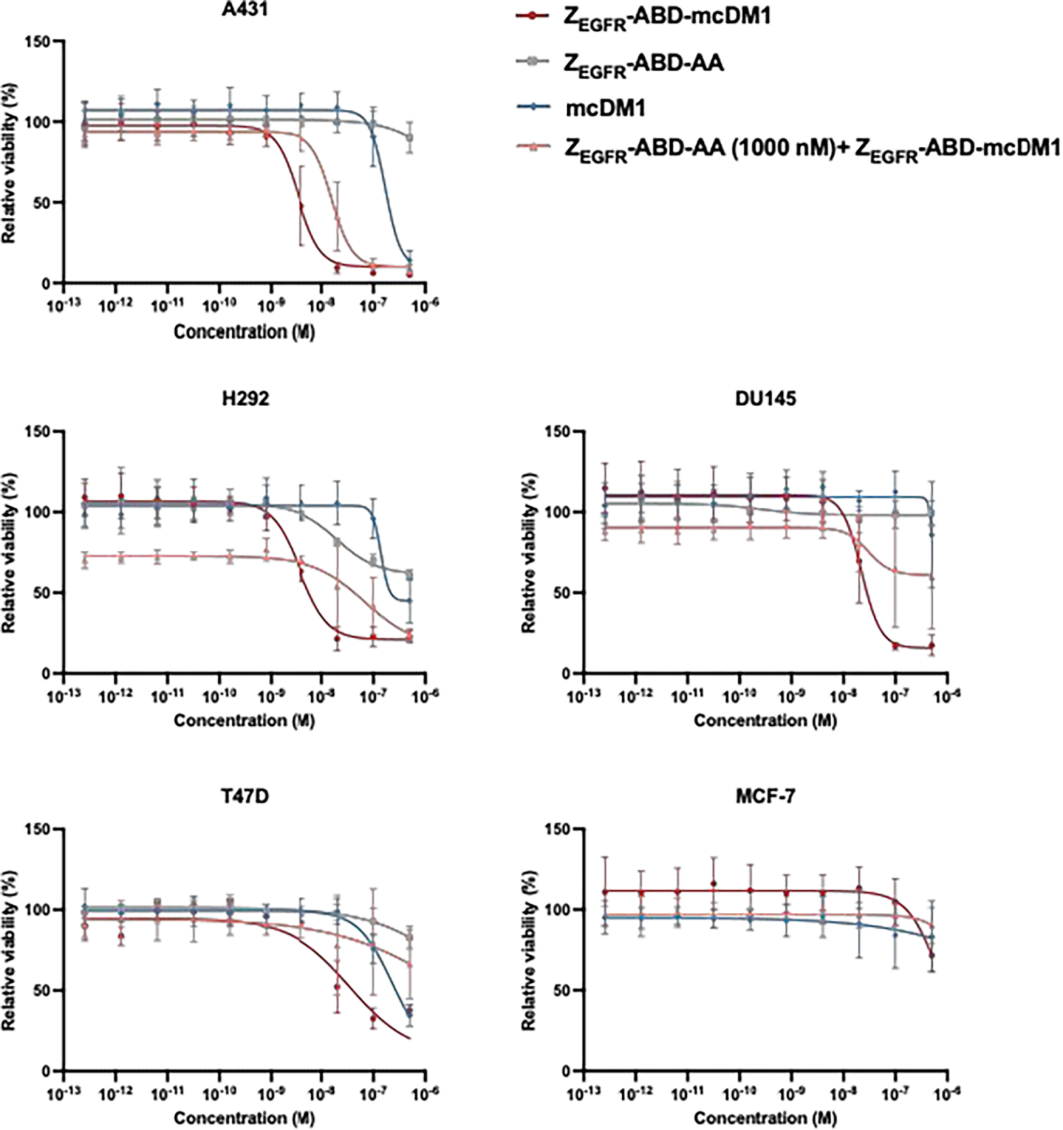
Cytotoxicity of the constructs. The cell lines indicated were treated with dilution series of Z_EGFR_-ABD-mcDM1, Z_EGFR_-ABD-AA, or mcDM1 for 72 h at 37 °C. In the blocking experiment, available EGFR was blocked by a large excess of Z_EGFR_-ABD-AA prior to the addition of Z_EGFR_-ABD-mcDM1. The viabilities were measured and normalized against cells grown without the addition of any construct, which was set to 100%. Each data point shows the mean of three independent experiments with error bars corresponding to 1 SD.

The nontoxic control Z_EGFR_-ABD-AA had a cytostatic effect at the highest concentrations. This effect was the most pronounced for H292, with a weaker effect for DU145. The viability of the other three cell lines was only slightly affected by Z_EGFR_-ABD-AA.

To further analyze the EGFR-dependence for the cytotoxic effect, available EGFR on the cell lines was blocked by preincubation with Z_EGFR_-ABD-AA (1000 nM) before the addition of dilution series Z_EGFR_-ABD-mcDM1. As can be seen in Figure 3, the IC_50_ values were substantially higher (weaker cytotoxic effect) in this setup, corroborating that EGFR-engagement is essential for efficient cell killing.

### 3.5 Radiolabeling and Stability

To allow for the determination of cellular interactions, internalization, and *in vivo* biodistribution, the constructs were labeled with 99m-Technetium. The labeling yields for [^99m^Tc]­Tc-Z_EGFR_-ABD-mcDM1 and [^99m^Tc]­Tc-Z_EGFR_-ABD-AA were 89 ± 6% and 87 ± 5%, respectively. The presence of impurities in the form of reduced hydrolyzed technetium colloids (RHT) was less than 1% for both constructs. The radiochemical purity was determined by ITLC following size exclusion purification and was found to be >99.9% for both radioconjugates. To test the stability of the radiolabel, the radioconjugates were incubated with a large excess of histidine (Table 3). Within the observation period of 24 h, a maximum of 4% release was observed, showing robust radiolabeling.

**Table 3:** Stability of the Radiolabel^a^

	[^99m^Tc]Tc-Z_EGFR_-ABD-mcDM1			[^99m^Tc]Tc-Z_EGFR_-ABD-AA		
	1 h	4 h	24 h	1 h	4 h	24 h
PBS	100 ± 0	100 ± 0	100 ± 0	100 ± 0	100 ± 0	100 ± 0
histidine, room temperature	99.3 ± 0.3	99.8 ± 0.2	98.8 ± 0.3	99.97 ± 0.06	99.4 ± 0.06	99.3 ± 0.5
histidine, 37 °C	99.8 ± 0.4	99.0 ± 0.9	97.5 ± 0.4	99.5 ± 0.6	97.5 ± 0.5	96.0 ± 0.8

a The radiolabeled constructs (1 μg each) were incubated in PBS or with a 1000-fold molar excess of histidine in PBS at room temperature or at 37°C. The amount of protein-associated radioactivity was determined by ITLC. The data is presented as the percent of protein-associated activity ± 1 SD (*n* = 3).

### 3.6 Cellular Binding, Specificity, Internalization, and Retention

Next, the binding specificity of [^99m^Tc]­Tc-Z_EGFR_-ABD-mcDM1 and [^99m^Tc]­Tc-Z_EGFR_-ABD-AA to four cancer cell lines with different expression levels of EGFR was analyzed (Figure 4A,B). The cells were incubated with the radioconjugates directly or after preincubation with a large excess of cetuximab, which competes with Z_EGFR_ for binding to EGFR. Preincubation of the cells with cetuximab resulted in a significant decrease in the percentage of cell-associated activity for all cell lines and for both constructs, showing that the interaction of the radioconjugates with the cells is blockable, which strongly suggests EGFR-specific binding to the cells. The percentage of cell-associated activity was the highest for A431 cells, followed by H292 cells. The percentage of cell-associated activity for DU145 and PC-3 cells was clearly lower than for the A431 and H292 cells.

**Figure 4:**
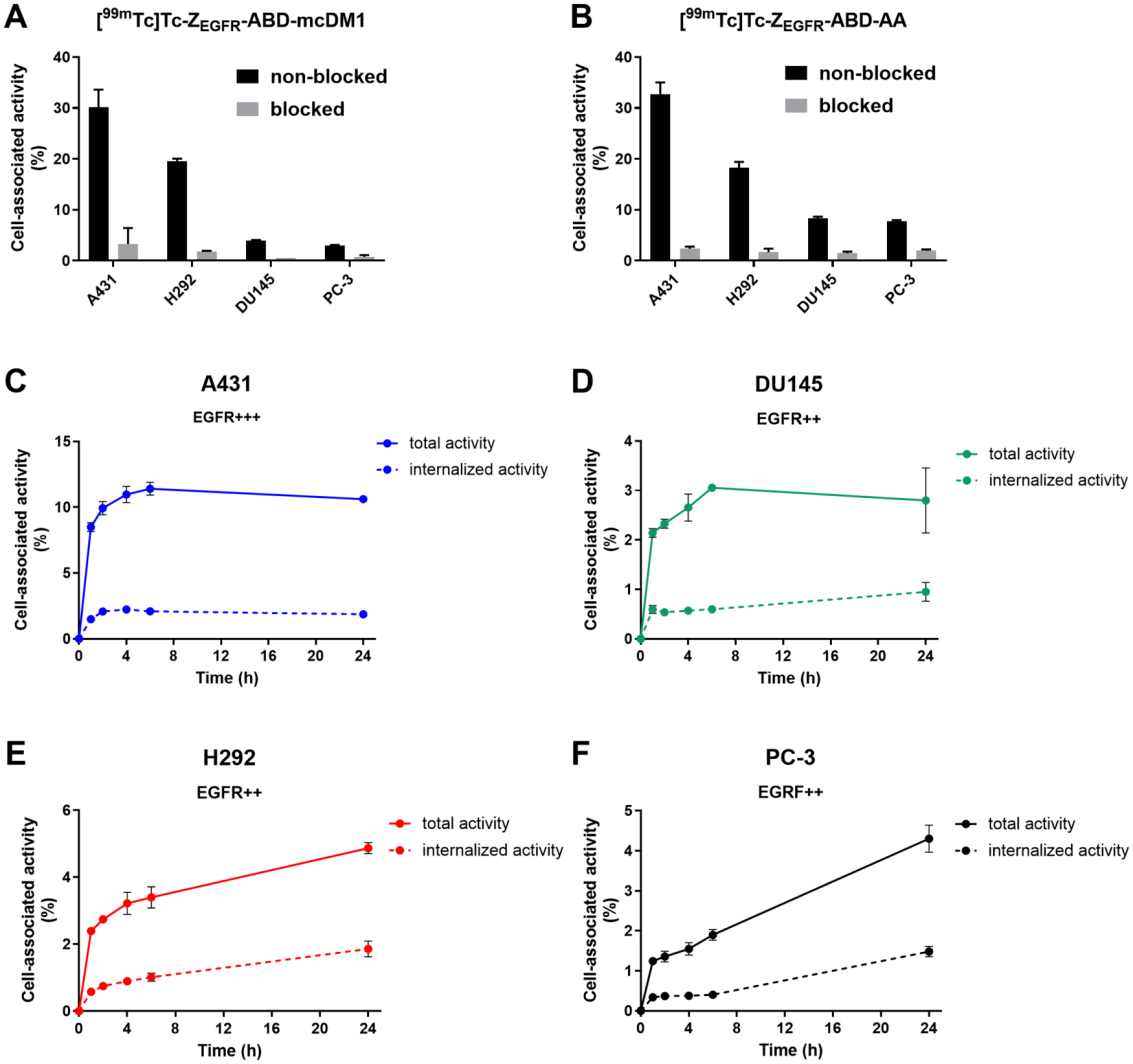
*In vitro* specificity of (A) [^99m^Tc]­Tc-Z_EGFR_-ABD-mcDM1 and (B) [^99m^Tc]­Tc-Z_EGFR_-ABD-AA to a panel of EGFR-expressing cell lines. In the blocked groups, cells were preincubated with 1000 nM of cetuximab prior to the addition of 10 nM of the [^99m^Tc]­Tc-labeled constructs. (C–F) Association and internalization of [^99m^Tc]­Tc-Z_EGFR_-ABD-mcDM1 by the EGFR-expressing cell lines A431 (C), DU145 (D), H292 (E) and PC-3 (F). The cells were continuously incubated with 1 nM of [^99m^Tc]­Tc-Z_EGFR_-ABD-mcDM1. At selected time points, the cell-associated activity was separated into membrane-bound and internalized fractions using the ‘acid wash’ method. The error bars correspond to 1 SD (*n* = 3).

To study the rate of internalization, A431, H292, DU145, and PC-3 cells were incubated with [^99m^Tc]­Tc-Z_EGFR_-ABD-mcDM1, and the cell-bound and internalized fractions were quantified. The results are displayed in Figure 4C–F. The association of [^99m^Tc]­Tc-Z_EGFR_-ABD-mcDM1 to the cells was generally quick, with >50% of the maximum cell-bound activity already after 1 h of incubation for all cell lines, except for PC-3, where the association was slower. For A431 cells, the cell-bound activity and internalized activity reached a plateau after 6 h of incubation. For H292, DU145 and PC-3 cells, the fraction of internalized activity continued to increase during the entire observation period.

### 3.7 Affinity to A431 Cells

Next, the interaction between the constructs and A431 cells, with high EGFR expression, was studied in real-time on a LigandTracer instrument. The derived kinetic parameters and equilibrium dissociation constants (*k*_a_, *k*_d_, and *K*_D_) are presented in Table 4. No significant differences between the kinetic constants or the equilibrium dissociation constants were observed for the two constructs.

**Table 4:** Interaction with A431 Cells^a^

	[^99m^Tc]Tc-(HE)_3_-Z_EGFR_-ABD-mcDM1	[^99m^Tc]Tc-(HE)_3_-Z_EGFR_-ABD-AA
*k*_a_ (1/Ms)	5.1 × 10^4^ ± 4.1 × 10^4^	3.4 × 10^4^ ± 1.0 × 10^4^
*k*_d_ (1/s)	9.8 × 10^–6^ ± 1.9 × 10^–6^	1.2 × 10^–5^ ± 0.7 × 10^–5^
*K*_D_ (pM)	290 ± 190	370 ± 200

a The interactions between A431 cells and ^[99m^Tc]­Tc-ZEGFR-ABD-mcDM1 and [^99m^Tc]­Tc-ZEGFR-ABD-AA were measured. The data are shown as the average ± 1 SD (*n* = 3).

### 3.8 Biodistribution and *In Vivo* Specificity

To investigate EGFR-targeting specificity *in vivo* and the overall biodistribution profile, including off-tumor uptake, [^99m^Tc]­Tc-Z_EGFR_-ABD-mcDM1 was injected in BALB/c nu/nu mice bearing A431 or RAMOS xenografts. The tumor uptake and biodistribution were determined at 4 and 24 h and are presented in Figure 5 and Table S1. At 4 h pi, the concentration in blood was 10 ± 1%IA/g, which significantly decreased with time to 3.5 ± 0.6%IA/g at 24 h, suggesting a terminal half-life in vivo of around 13 h. The tumor uptake was 2.2 ± 0.5%IA/g 4 h pi and did not significantly change from 4 to 24 h, though a tentative increase could be observed. At both time points, the kidneys and the liver were the normal organs with the highest activity uptake. The uptake in kidneys and liver decreased significantly from 4 to 24 h pi, as well as the uptake in the gastrointestinal tract (GI) and body. Comparing the uptake of [^99m^Tc]­Tc-Z_EGFR_-ABD-mcDM1 in EGFR-positive A431 xenografts with the uptake in EGFR-negative RAMOS xenografts (Figure 5B), a significantly higher uptake in the A431 xenografts was observed, indicating EGFR-specific interaction of [^99m^Tc]­Tc-Z_EGFR_-ABD-mcDM1 *in vivo*.

**Figure 5:**
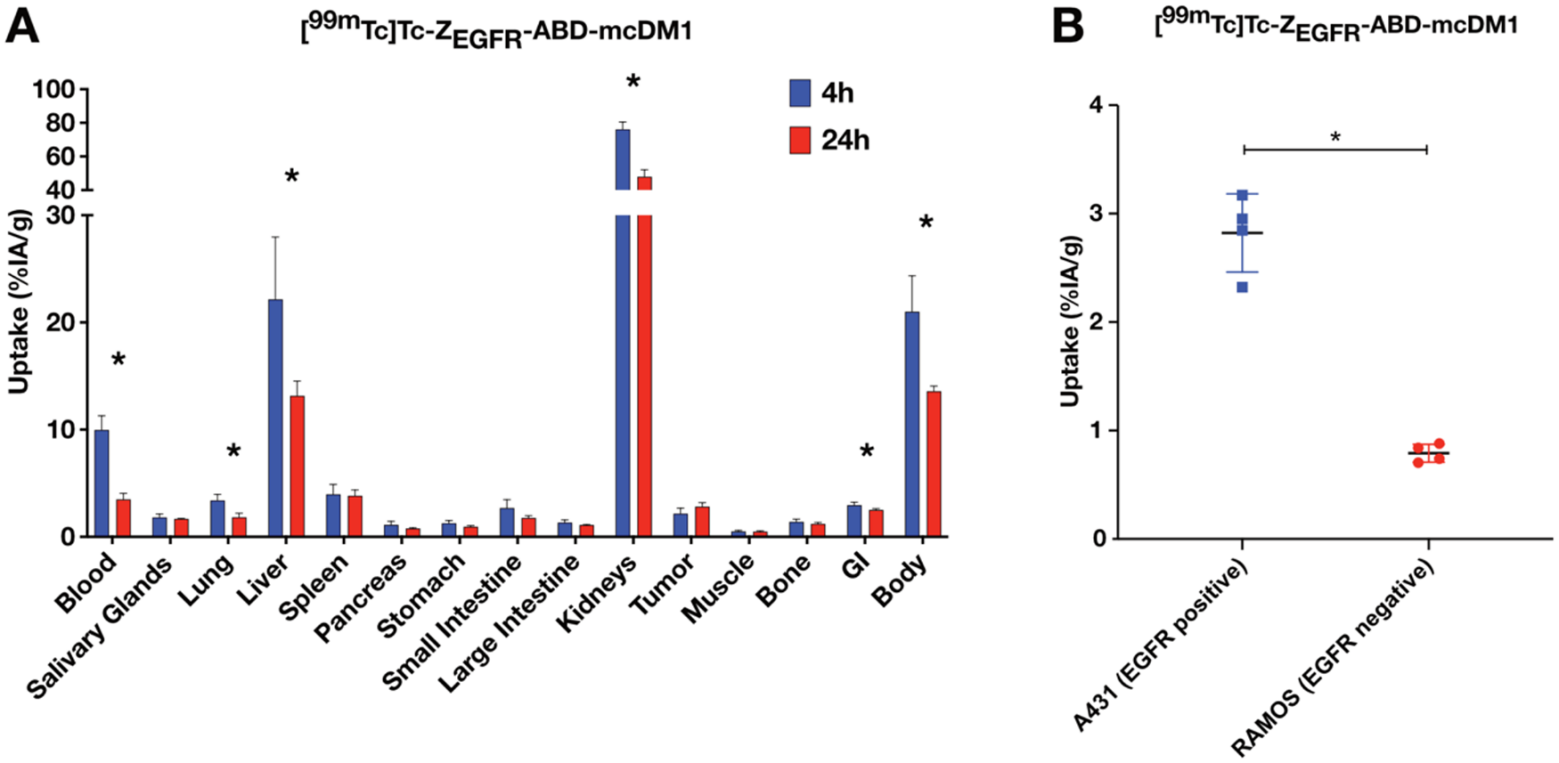
(A) Biodistribution of [^99m^Tc]­Tc-Z_EGFR_-ABD-mcDM1 in female BALB/c nu/nu mice bearing EGFR-expressing A431 xenografts. The uptake is presented as the average ± 1 SD (*n* = 4) of %IA/g. The numbers for gastrointestinal tract (GI) and body are presented as %IA per whole sample. *Indicate a statistically significant difference (*p* < 0.05) between the 4 and 24 h time points. (B) *In vivo* specificity of [^99m^Tc]­Tc-Z_EGFR_-ABD-mcDM1. The uptake of [^99m^Tc]­Tc-Z_EGFR_-ABD-mcDM1 in EGFR-positive A431 xenografts was compared with the uptake in EGFR-negative RAMOS xenografts at 24 h pi. *Indicate a statistically significant difference (*p* < 0.05) in uptake between the A431 and RAMOS xenografts.

## Discussion

The epidermal growth factor receptor is overexpressed in a number of cancers,^9^ and drugs targeting this receptor thus have the potential to be of great clinical benefit to many patients. Small-molecule inhibitors targeting EGFR are approved for therapy, but resistance develops for nearly all patients with time.^3^ Protein-based drugs show some clinical benefit, but resistance develops to them as well. We have therefore investigated a novel modality of an EGFR-targeted drug, a so-called affibody drug conjugate (AffiDC) consisting of an EGFR-targeting affibody carrying a highly cytotoxic DM1 molecule.

The newly designed AffiDC, Z_EGFR_-ABD-mcDM1, could be easily produced to a high purity. The primary characterization by RP-HPLC and size-exclusion chromatography showed monodisperse constructs with a purity of >99%. Unlike antibody-based drugs, the fusion protein Z_EGFR_-ABD could be produced in a simple prokaryotic host cell, in this case *Escherichia coli*, which offers a cost-effective approach for large-scale production.^45^ Furthermore, in contrast to antibodies, the affibody framework does not contain any cysteines. During the design of AffiDCs, this allows for the insertion of one or more cysteines, which can be specifically addressed by maleimide-functionalized cytotoxic drugs, such as mcDM1. It gives firm control of the site of attachment as well as the drug-to-antibody/affibody ratio (DAR). In Z_EGFR_-ABD-mcDM1, a single cysteine was inserted in the C-terminus and was used for DM1 conjugation. It was positioned away from the EGFR-binding site of the affibody so that DM1 conjugation would not affect the interaction with EGFR. Indeed, affinity measurements showed that neither ABD fusion nor DM1 conjugation affected its affinity to human or murine EGFR, which was 1–2 nM at 25 °C in this study, compared to 0.8 nM for the affibody by itself to human EGFR in a previous study.^25^ The affinity of the ABD to serum albumin was slightly decreased by the conjugation of mcDM1; the affinity of Z_EGFR_-ABD-mcDM1 and Z_EGFR_-ABD-AA for HSA was 3.2 and 0.7 nM, respectively. An interesting observation during the biochemical characterization of the constructs was that they were eluted slightly earlier in the size-exclusion chromatography analysis than their molecular weight would suggest (Figure 1C). Size-exclusion chromatography relies on both size and shape for separation, and unlike the protein standards, which are globular, the constructs are not globular but consist of two independently folding domains. A plausible explanation is therefore that their different shape partially prevents them from entering the pores of the beads in the size-exclusion matrix, and they are therefore eluted slightly earlier.

Z_EGFR_-ABD-mcDM1 was highly cytotoxic to the A431 cell line, expressing a high level of EGFR. The IC_50_ value was 3.4 nM, which is similar to the IC_50_ values for HER2-targeting AffiDCs to SKBR3 and AU565 cells, both of which express a high level of HER2.^33^ The IC_50_ value is also comparable to the EGFR-targeted antibody-drug conjugate, losatuxizumab vedotin, on A431 cells (1 nM), an ADC that showed a promising effect in a phase I clinical trial.^18^ Furthermore, the EGFR-targeted antibody 40H3 was functionalized with different cytotoxic drugs, and the best-performing variant, 40H3-Tesirine, had an *in vitro* IC_50_ value of 1.8 nM on A431 cells and performed well in an A431 xenograft mouse model.^46^ It should be noted that losatuxizumab vedotin and 40H3-Tesirine use different linkers and cytotoxic drugs which makes a direct comparison with Z_EGFR_-ABD-mcDM1 not possible. However, an IC_50_ value *in vitro* of around 1 nM to A431 cells appears to be sufficiently potent for a response in a xenograft model in mice as well as a therapeutic response in patients. Future studies on Z_EGFR_-ABD-mcDM1 in xenograft models, and if successful, followed by clinical trials, are therefore motivated. Moreover, in a recent publication by Yang and co-workers, Z_EGFR:1907_ was loaded with mc-VC-PAB-MMAE followed by assembly into a nanoagent.^47^ Z_EGFR:1907_ is a variant of the affibody used in the present study. The nanoagent was similarly cytotoxic in vitro (IC_50_ 4.8 nM) to the A431 cell line, compared to Z_EGFR_-ABD-mcDM1 (IC_50_ 3.4 nM). Interestingly, it could be used to efficiently treat mice with implanted A431-derived tumors, which motivates further evaluation of Z_EGFR_-ABD-mcDM1 in preclinical mouse models of human cancer in the future.

The drug conjugates could be stably labeled with 99m-Technetium on the N-terminal (HE)_3_-tag, as shown by the histidine challenge test. The radiometal 99m-Technetium in a tricarbonyl complex (Tc­(CO)_3_^+^) exhibits residualizing properties after cell internalization and the measured activity value for an organ at a given time-point, therefore, should reflect the total uptake of the conjugate up until that point.

Of importance when designing drug conjugates is to avoid creating too hydrophobic constructs that may interact with cells and organs nonspecifically. For example, in a study by Hamblett and co-workers,^48^ an anti-CD30 ADC with different DARs was generated, and the construct with a drug load of eight had a significantly lower therapeutic index in mice compared to a construct with a drug load of two or four. For smaller AffiDCs targeting HER2, increasing the drug load from one to three was similarly found to result in a significantly more hydrophobic construct with increased liver uptake *in vivo*.^49^ In this study, we, therefore, chose a drug load of one, and the resulting drug conjugate was found to interact in a receptor-specific (blockable) manner with EGFR-expressing cells (Figure 4A,B). Only a minor interaction with the cells was detected after blocking with cetuximab, showing that the unspecific interaction with the cells was minor.

EGFR is activated by a variety of ligands, which in turn trigger internalization via homo- or heterodimerization with other members of the ErbB-family.^50^ The rate of internalization was shown to differ dramatically between the cell lines, where the internalized fraction of [^99m^Tc]­Tc-Z_EGFR_-ABD-mcDM1 by H292, PC-3, and DU145 cells was twice as high after 24 h compared to the A431 cells. However, it is important to note that Figure 4C–F show the internalized fraction normalized to the total cell-bound activity, and since the cell-bound activity is lower for H292, PC-3, and DU145 than for the A431 cell line (Figure 4A,B), the internalized number of AffiDC-molecules is higher for A431 cells. It is therefore not surprising that the A431 cell line, having the highest number of receptors, was the most sensitive to the AffiDC’s cytotoxic action, despite having the lowest internalized fraction. A431 was also the most sensitive to free mcDM1 (Figure 3). It should also be noted that other parameters may play a role in cell lines’ sensitivity to drug conjugates, such as defective intracellular transport and lysosomal degradation, as well as the upregulated expression of multidrug resistance proteins.^51^ Differences in sensitivity to drug conjugates between different cell lines, having a similar expression level of the targeted receptor, have been observed by us and others.^52,53^

The biodistribution experiment showed medium activity uptake in the A431-derived tumors with relatively high uptake in the liver and kidneys. The rest of the organs had only low activity uptake. Furthermore, the biodistribution experiment showed a significantly higher uptake in EGFR-expressing A431 xenografts than in EGFR-negative RAMOS xenografts, demonstrating that tumor uptake was EGFR-dependent, and that the uptake mediated by the enhanced permeability and retention (EPR) effect was only minor. Compared to an earlier study on [^99m^Tc]­Tc-Z_EGFR:2377_,^54^ utilizing the same affibody, but lacking the ABD and DM1, some interesting observations were made. While the tumor uptake was similar, the clearance route shifted more toward the liver for [^99m^Tc]­Tc-Z_EGFR_-ABD-mcDM1, which could be a consequence of the hydrophobic DM1 molecule, as well as a longer half-life mediated by the ABD. The initial renal activity uptake was almost 2.5-fold lower for [^99m^Tc]­Tc-Z_EGFR_-ABD-mcDM1 compared to the non-ABD-fused [^99m^Tc]­Tc-Z_EGFR:2377_, while the hepatic activity uptake increased over 4-fold. The observed shift in the excretion route is in agreement with earlier studies on ABD-fused affibody molecules interacting with other targets: HER2, HER3 and VEGFR-2.^55–57^ The activity uptake in tumors demonstrated a tendency to increase from the 4 to 24 h time points in this study for the ABD-fused conjugate, while the activity uptake decreased for [^99m^Tc]­Tc-Z_EGFR:2377_ for the same time interval.^54^ It is likely a consequence of the increased half-life mediated by the ABD, and a comparison of the activity concentration in blood showed that it was about 5-fold higher for [^99m^Tc]­Tc-Z_EGFR_-ABD-mcDM1 than for [^99m^Tc]­Tc-Z_EGFR:2377_ at 4 h and about 10-fold higher at 24 h.

Clearly, a low liver uptake is desirable, particularly for EGFR-targeted drugs, since there is endogenous EGFR-expression in the liver. An appealing approach could be to employ a prodrug concept, where the EGFR paratope on the affibody is masked until it enters the tumor, where it is activated. We have earlier investigated a prodrug-affibody format, where an anti-idiotypic affibody was coupled to Z_EGFR_ by a linker that could be cleaved by the tumor-excreted protease, matriptase, to activate Z_EGFR_ binding.^58^ Since nontumor cells do not excrete matriptase, binding to EGFR on normal organs is minimized. In the study by Dahlsson Leitao and co-workers,^58^ the liver uptake was decreased by 50% when employing the pro-affibody format. It could be a promising format also for EGFR-targeted drug conjugates in the future.

In summary, we have investigated an affibody-based drug conjugate targeting EGFR, Z_EGFR_-ABD-mcDM1. Collectively, the results show a potent and EGFR-specific drug conjugate that holds promise for further preclinical and potentially clinical development.

## Supplementary Material



## Data Availability

Data will be made available on request.

## Notes

Liquid chromatography coupled electrospray ionization quadrupole time-of-flight (ESI-qTOF) mass spectrometry analysis of the constructs (Figure S1); surface plasmon resonance analysis of the interaction between the constructs and human EGFR (Figure S2); surface plasmon resonance analysis of the interaction between the constructs and mouse EGFR (Figure S3); surface plasmon resonance analysis of the interaction between the constructs and serum albumin (Figure S4); surface plasmon resonance analysis of the interaction between the constructs and HER2 (Figure S5); analysis of relative EGFR expression on different cell lines (Figure S6); biodistribution of [^99m^Tc]­Tc-Z_EGFR_-ABD-mcDM1 in BALB/c nu/nu mice bearing A431 or RAMOS xenografts at 4 and 24 h (PDF)

## Abbreviations

ABD: albumin-binding domain
ADC: antibody drug conjugate
AffiDC: affibody drug conjugate
BC: breast cancer
CD: circular dichroism
CRC: colorectal cancer
DAR: drug to antibody/affibody ratio
EGF: epidermal growth factor
EGFR: epidermal growth factor receptor
EPR: enhanced permeability and retention
GBM: glioblastoma multiforme
GI: gastrointestinal tract
HER2: human epidermal growth factor receptor 2
HNSCC: head and neck squamous cell carcinoma
HSA: human serum albumin
IC_50_: half maximal inhibitory concentration
ITLC: instant thin layer chromatography
*k*_a_: association rate
*k*_d_: dissociation rate
*K*_D_: equilibrium dissociation constant
mAb: monoclonal antibody
MAPK: mitogen-activated protein kinase
mc: maleimidocaproyl
MSA: mouse serum albumin
NSCLC: nonsmall cell lung cancer
nTPM: normalized transcript expression value
PBS: phosphate buffered saline
RHT: reduced hydrolyzed technetium colloids
RP-HPLC: reversed-phase high-performance liquid chromatography
TFA: trifluoroacetic acid
T-DM1: trastuzumab emtansine
TKI: tyrosine kinase inhibitor
VEGFR-2: vascular endothelial growth factor receptor 2
