# Supplementary material for: Affibody-Derived Drug Conjugates Targeting The Epidermal Growth Factor Receptor Are Potent And Specific Cytotoxic Agents
Source: ACS Pharmacol Transl Sci. 2025 Oct 31;8(11):3872–85. doi: 10.1021/acsptsci.5c00079 (PMC12624491; doi:10.1021/acsptsci.5c00079)
Supplement: Supplementary file 1 [file pt5c00079_si_001.pdf]

Supporting Information

Affibody-derived drug conjugates targeting the epidermal growth factor receptor are potent and specific cytotoxic agents

Sara S. Rinne[1†], Wen Yin[2†], Ruonan Li[2†], Haozhong Ding[2], Anna Mestre Borras[2], Chenar Mahmod[1], Stefan Ståhl[2], Anna Orlova[1], John Löfblom[2], Anzhelika Vorobyeva[3], Torbjörn Gräslund[2]*

[1]Department of Medicinal Chemistry, Uppsala University, 751 23 Uppsala, Sweden.

[2]Department of Protein Science, KTH Royal Institute of Technology, Roslagstullsbacken 21, 106 91 Stockholm, Sweden.

[3]Department of Immunology, Genetics and Pathology, Uppsala University, Dag Hammarskjölds väg 20, 751 85 Uppsala, Sweden.

*Correspondence: torbjorn@kth.se

[†] These authors contributed equally to this work.

Table of contents

Supplementary figures 3
Supplementary tables 9

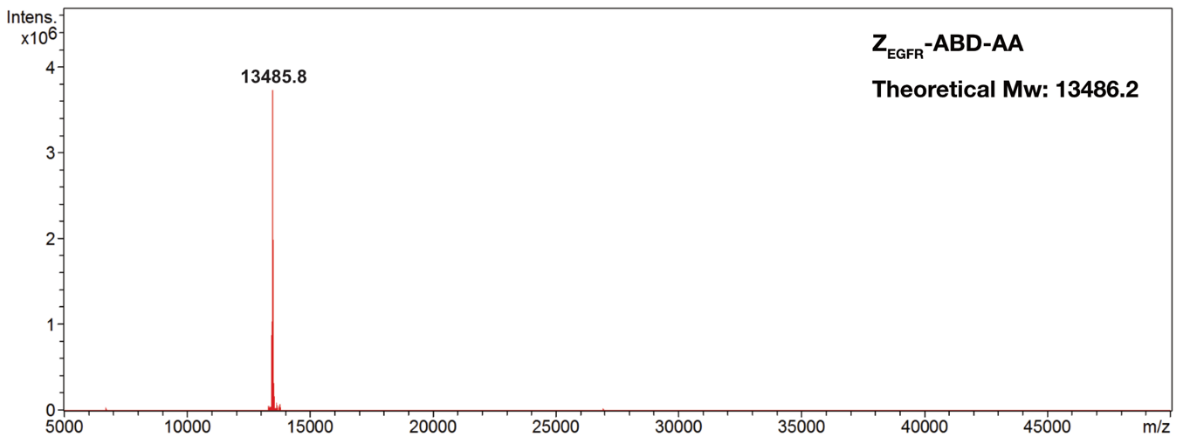

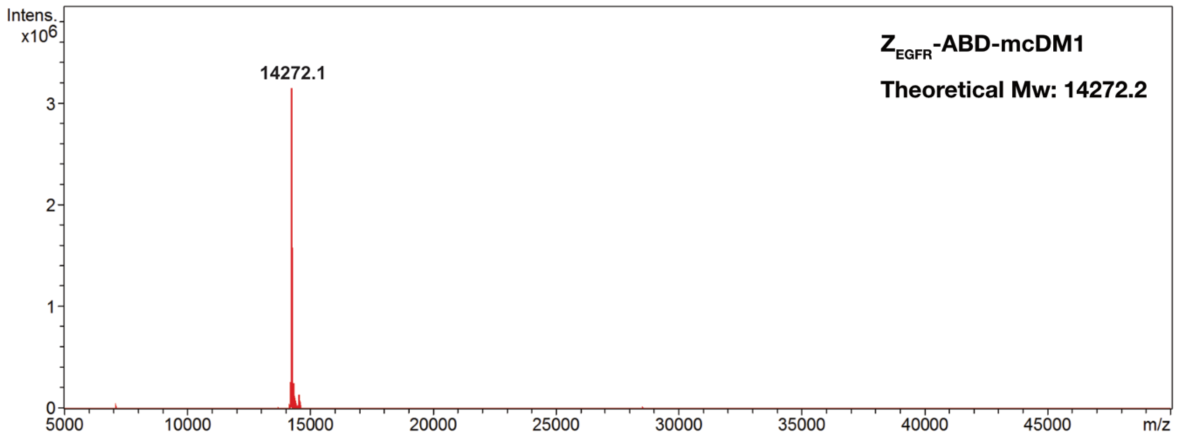

Figure S1. Z_{EGFR}-ABD-mcDM1 and Z_{EGFR}-ABD-AA were subjected to liquid chromatography coupled electrospray ionization quadrupole time-of-flight (ESI-qTOF) mass spectrometry. The spectra show the recorded intensities as a function of the mass-to-charge ratio. The measured molecular weights agree within 1 Da from the theoretical values.

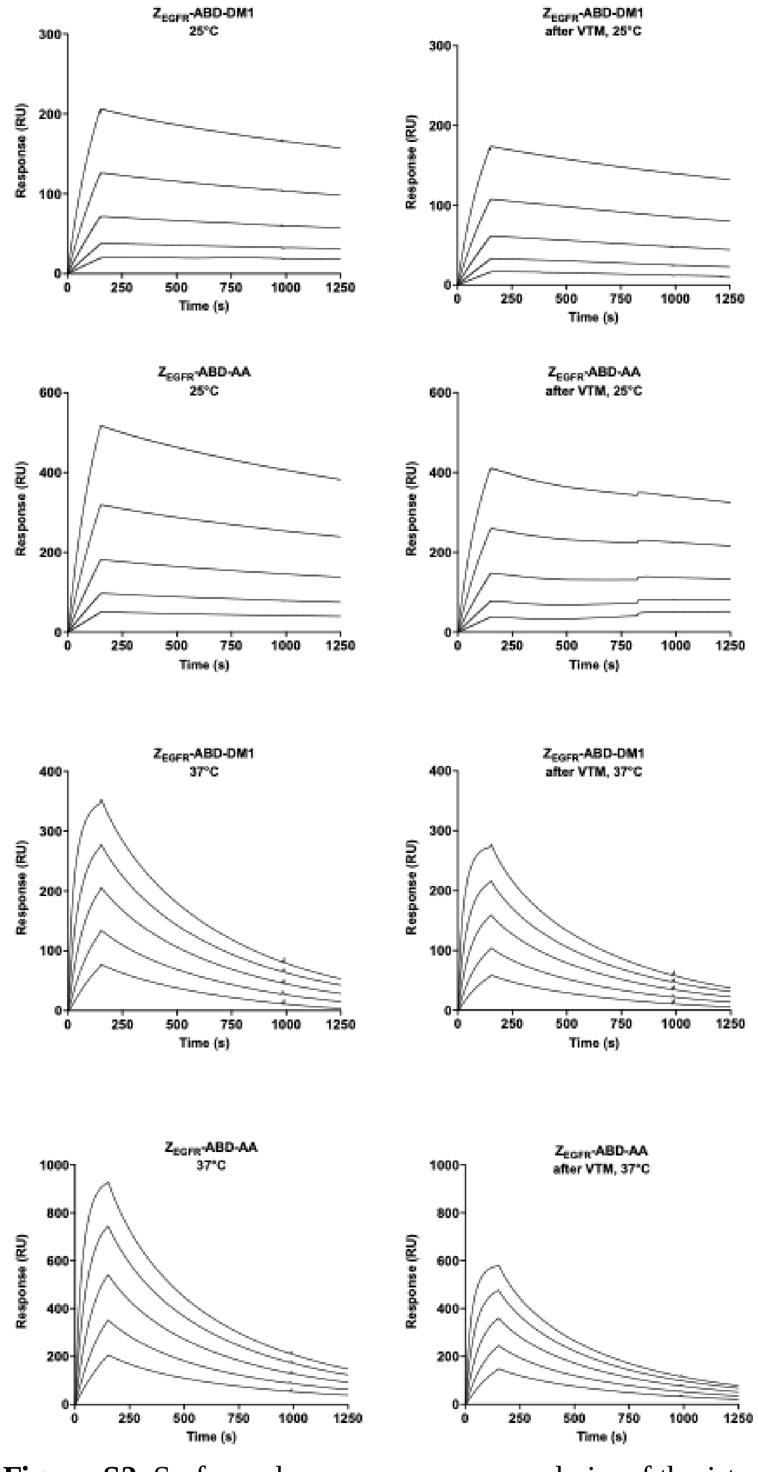

Figure S2. Surface plasmon resonance analysis of the interaction between, Z_{EGFR}-ABD-DM1 and Z_{EGFR}-ABD-AA, and human EGFR. Z_{EGFR}-ABD-DM1 and Z_{EGFR}-ABD-AA (100 nM) was injected over flowcells with immobilized HSA (sensorgram removed). Subsequently, two-fold dilution series of hEGFR (3.125-100 nM at 37°C; 3.125-50 nM at 25°C) were injected in serial-mode over the flowcells. Each injection was done in duplicates. The panel shows an overlay of the sensorgrams obtained for each interaction, where the differences in the duplicate sensorgrams might be difficult to distinguish. The construct and temperature are indicated above each panel. VTM is an abbreviation for variable temperature measurement, i.e. indicates material that has been thermally denaturated and refolded.

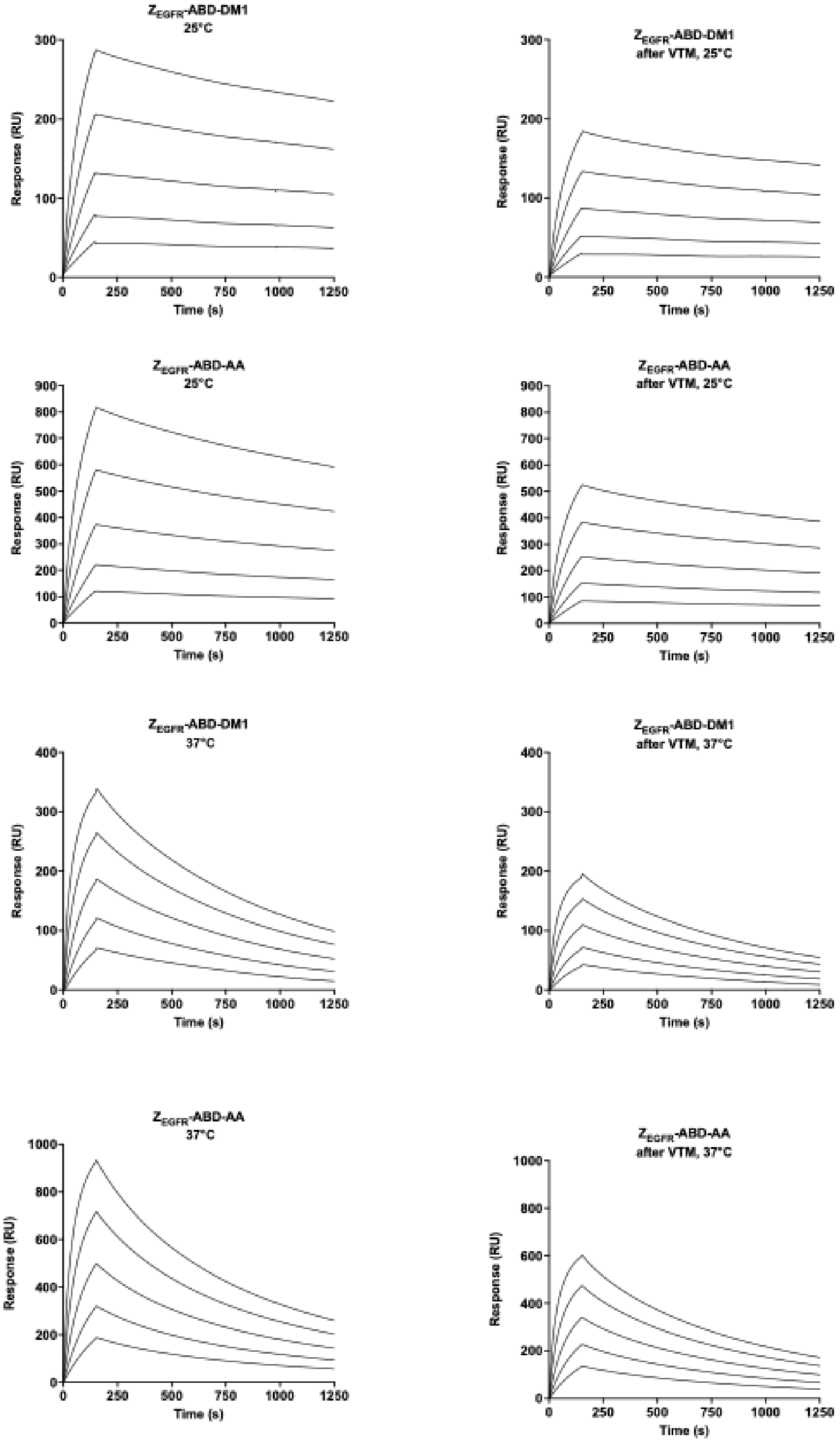

Figure S3. Surface plasmon resonance analysis of the interaction between Z_{EGFR}-ABD-DM1 and Z_{EGFR}-ABD-AA, and murine EGFR. Z_{EGFR}-ABD-DM1 and Z_{EGFR}-ABD-AA (100 nM) were injected over flow cells with immobilized HSA (sensorgram removed). Subsequently, two-fold dilution series of mEGFR (3.125-100 nM) were injected in serial mode over the flow cells. Each injection was done in duplicate. The panel shows an overlay of the sensorgrams obtained for each interaction, where the differences in the duplicate sensorgrams might be difficult to distinguish. The construct and temperature are indicated above each panel. VTM is an abbreviation for variable temperature measurement, i.e. indicates material that has been thermally denaturated and refolded.

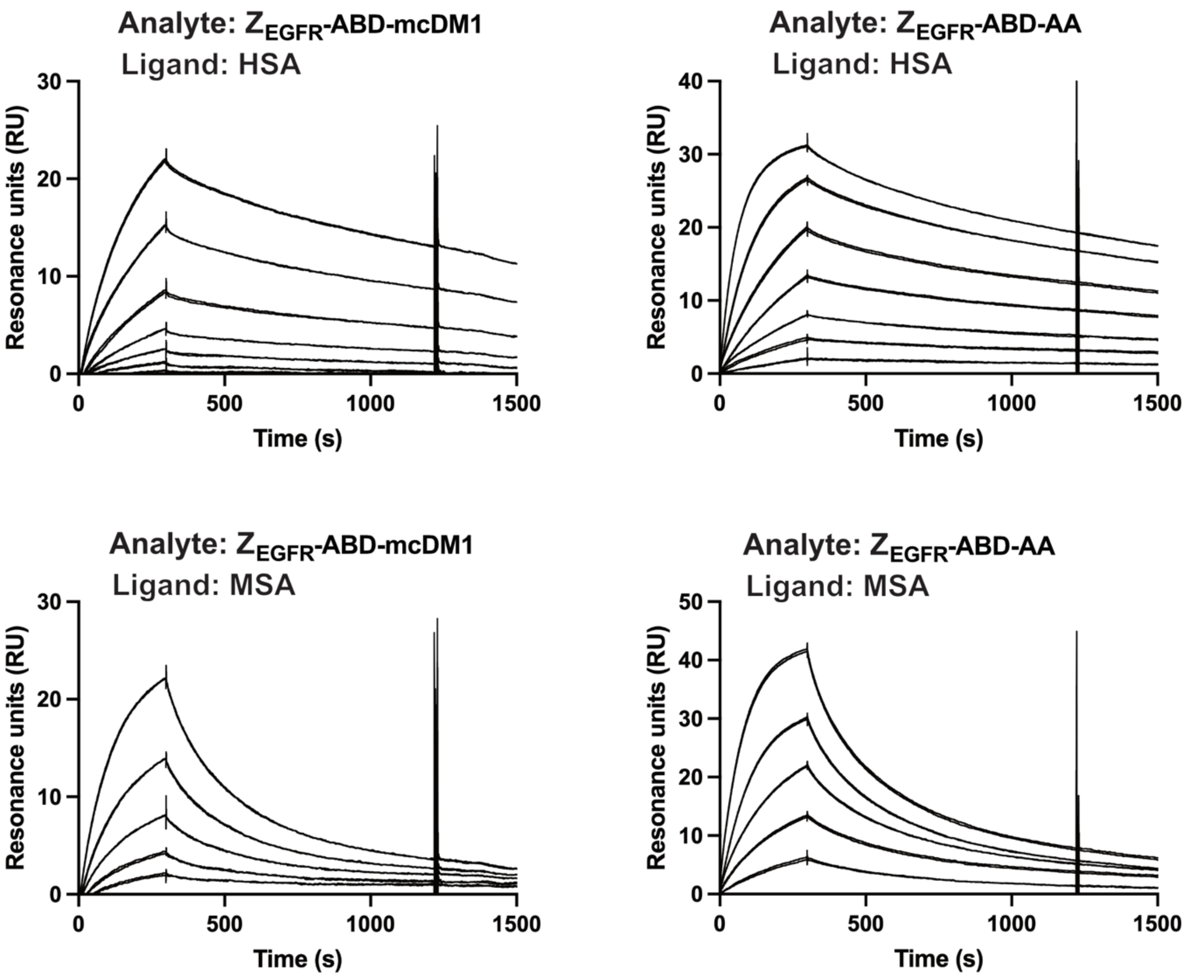

Figure S4. Surface plasmon resonance analysis of the interaction between the Z_{EGFR}-ABD-DM1 and Z_{EGFR}-ABD-AA, and serum albumins. A two-fold dilution series (0.4-25 nM) of each construct was prepared and injected over a chip surface with immobilized HSA. A two-fold dilution series (0.4-6.3 nM) of each construct was prepared and injected over a chip surface with immobilized MSA. Each injection was done in duplicate. The panels show an overlay of the sensorgrams recorded for each interaction.

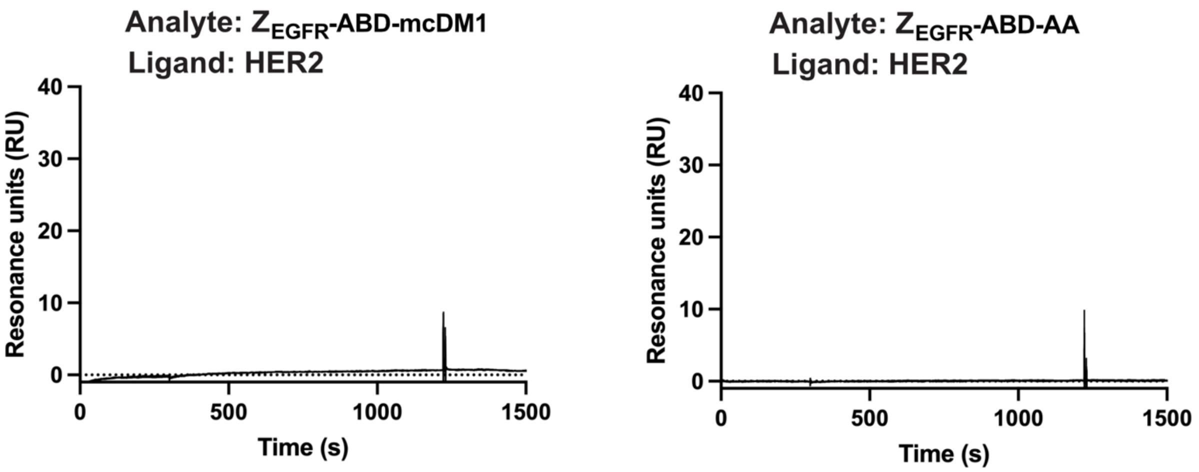

Figure S5. Surface plasmon resonance analysis of the interaction between HER2 and the constructs. HER2 was immobilized onto a sensor chip, after which dilution series (200, 100, and 50 nM) of Z_{EGFR}-ABD-DM1 and Z_{EGFR}-ABD-AA were sequentially injected over the chip in duplicates. The panels show an overlay of the six recorded sensorgrams for each interaction. As expected, no interaction was observed.

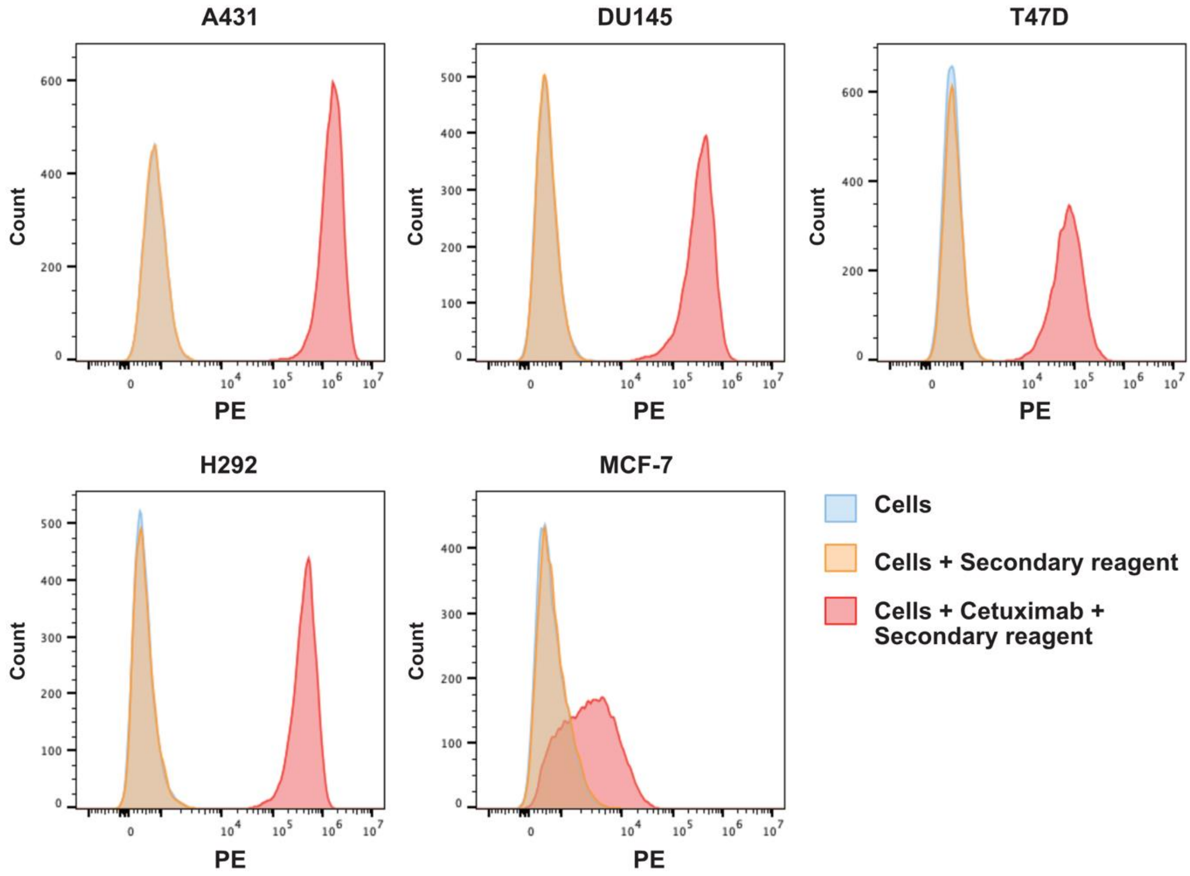

Supplementary Figure S6. Analysis of relative EGFR expression on different cell lines. Each panel shows an overlay of cells stained with the primary reagent cetuximab (human anti-EGFR mAb) followed by the secondary reagent (PE conjugated F(ab')2-Goat anti-Human IgG Fc gamma Secondary Antibody (Invitrogen)). As controls, unstained cells were analyzed (Cells) and cells stained with secondary reagent only (Cells + Secondary reagent). For all cell lines, staining was performed with 0.5 µg Cetuximab in 100 µL PBS supplemented with 2% Foetal Bovine Serum at room temperature for 45 min. After washing, the cells were incubated with secondary reagent, diluted 1:500 in PBS supplemented with 2% Foetal Bovine Serum, for 30 min on ice. The PE fluorescence is displayed on the x-axis with arbitrary units, and the cell count is displayed on the y-axis.

Table S1. Biodistribution of $[^{99m}\text{Tc}]\text{Tc-Z}_{\text{EGFR}}$-ABD-mcDM1 in BALB/c nu/nu mice bearing A431 or RAMOS xenografts at 4 and 24 h (n= 4).

**Organ**	**A431**		**RAMOS**
	4 h	24 h	24 h
Blood	10.0 ± 1.3	3.5 ± 0.6	4.0 ± 0.4
Salivary Glands	1.8 ± 0.3	1.7 ± 0.1	1.7 ± 0.1
Lung	3.4 ± 0.6	1.8 ± 0.4	2.0 ± 0.1
Liver	22.2 ± 5.8	13.2 ± 1.4	12.5 ± 1.5
Spleen	4.0 ± 0.9	3.8 ± 0.5	3.5 ± 0.8
Pancreas	1.1 ± 0.3	0.8 ± 0.1	0.8 ± 0.1
Stomach	1.3 ± 0.3	1.0 ± 0.1	1.0 ± 0.1
Small Intestine	2.7 ± 0.8	1.8 ± 0.2	2.1 ± 0.6
Large Intestine	1.3 ± 0.3	1.1 ± 0.1	1.1 ± 0.1
Kidneys	76.1 ± 4.4	48.0 ± 4.2	47.7 ± 1.7
Tumor	2.2 ± 0.5	2.8 ± 0.4	0.8 ± 0.1
Muscle	0.5 ± 0.1	0.5 ± 0.1	0.5 ± 0.1
Bone	1.4 ± 0.2	1.2 ± 0.1	1.2 ± 0.1
GI*	2.9 ± 0.3	2.5 ± 0.1	2.5 ± 0.2
Body*	21.0 ± 3.3	13.6 ± 0.5	14.7 ± 0.6
